# Post-transcriptional Regulation of Gene Expression via Unproductive Splicing

**DOI:** 10.32607/actanaturae.27337

**Published:** 2024

**Authors:** L. G. Zavileyskiy, D. D. Pervouchine

**Affiliations:** Lomonosov Moscow State University, Moscow, 119192 Russian Federation; Skolkovo Institute of Science and Technology, Moscow, 121205 Russian Federation

**Keywords:** unproductive splicing, nonsense-mediated decay, splicing, regulation, antisense oligonucleotides

## Abstract

Unproductive splicing is a mechanism of post-transcriptional gene expression control in which premature stop codons are inserted into protein-coding transcripts as a result of regulated alternative splicing, leading to their degradation via the nonsense-mediated decay pathway. This mechanism is especially characteristic of RNA-binding proteins, which regulate each other’s expression levels and those of other genes in multiple auto- and cross-regulatory loops. Deregulation of unproductive splicing is a cause of serious human diseases, including cancers, and is increasingly being considered as a prominent therapeutic target. This review discusses the types of unproductive splicing events, the mechanisms of auto- and cross-regulation, nonsense-mediated decay escape, and problems in identifying unproductive splice isoforms. It also provides examples of deregulation of unproductive splicing in human diseases and discusses therapeutic strategies for its correction using antisense oligonucleotides and small molecules.

## INTRODUCTION

Eukaryotic gene expression is controlled by a large number of factors that regulate a balance between mRNA synthesis and degradation [1, 2]. Nonsense mutations and frameshifting splicing errors lead to the emergence of mRNA isoforms containing premature termination codons (PTC). Eukaryotes have a system for selective degradation of such transcripts, called the nonsense-mediated decay (NMD) [3].

**Fig. 1:**
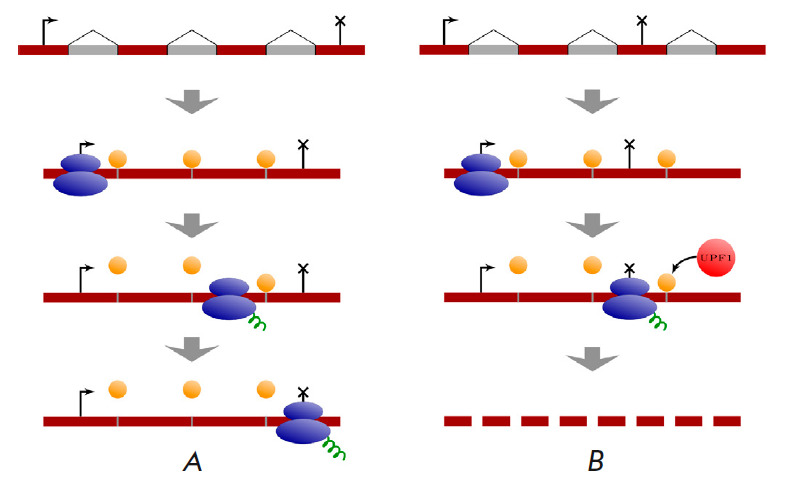
The EEJ-dependent mechanism of NMD. (*A*) EEJ complexes (orange circles) are displaced from the mRNA by the ribosome during the first round of translation. (*B*) The EEJ complexes that remain bound to mRNA outside of the reading frame serve as a signal that a PTC has appeared

It has long remained unclear how the NMD pathway recognizes PTCs and distinguishes them from the normal stop codons [4]. The current model suggests that PTC recognition occurs in the cytoplasm, with the participation of the exon–exon junction (EEJ) complexes that are deposited on pre-mRNA during splicing [5, 6]. After the first round of translation, EEJ proteins located within the reading frame are displaced from pre-mRNA by ribosome (*Fig. 1A*) [7, 8, 9]. Since the normal translation termination site is usually located in the last exon [10], the EEJ proteins that remain bound to the pre-mRNA outside of the reading frame serve as a signal that a PTC has appeared (*Fig. 1B*). The presence of an EEJ 50–55 or more nucleotides downstream of the stop codon activates a cascade of transcript degradation, the central role in which is played by the UPF1 protein. The phosphorylated form of this protein attracts the endonuclease SMG6 and other factors that cause deadenylation and removal of the 5’-cap in pre-mRNA, which, in turn, triggers transcript decay by cellular exonucleases [9, 11, 12, 13]. There are other models in which PTCs are determined by the distance to the poly(A) tail, as well as models in which PTC causes mRNA degradation independently of EEJ proteins [14, 15, 16, 17, 18]. The existence of an EEJ-independent NMD mechanism explains the presence of a large number of NMD targets despite the almost complete lack of splicing in yeast [19, 20].

The primary function of NMD was originally believed to consist in preventing the emergence of truncated and, therefore, deleterious proteins [21]. However, it has become increasingly evident that NMD is ubiquitously used by the cell to regulate gene expression levels [22, 23]. For example, many RNAbinding proteins (RBPs) employ NMD to control their own expression through a negative feedback loop in which the protein product binds to its cognate mRNA and induces alternative splicing (AS) that leads to the generation of a PTC [24, 25]. Many splicing factors cross-regulate each other’s expression levels in this way [26, 27]. The mechanism in which alternative splicing and NMD cooperate to post-transcriptionally regulate mRNA expression levels occurs in all known eukaryotes and is evolutionarily conserved [26, 28]. In the literature, it is referred to as regulated unproductive splicing and translation (RUST) or simply unproductive splicing [22, 29].

## TYPES OF UNPRODUCTIVE SPLICING

Regulated transcript degradation through NMD depends on alternative splicing (AS), in which multiple mature mRNA isoforms are generated from the same pre-mRNA. AS events are usually categorized into few simple classes, such as exon skipping, alternative 5’- or 3’-splice sites, intron retention, mutually exclusive exons, but there are also more complex types of AS events [30, 31].

**Fig. 2:**
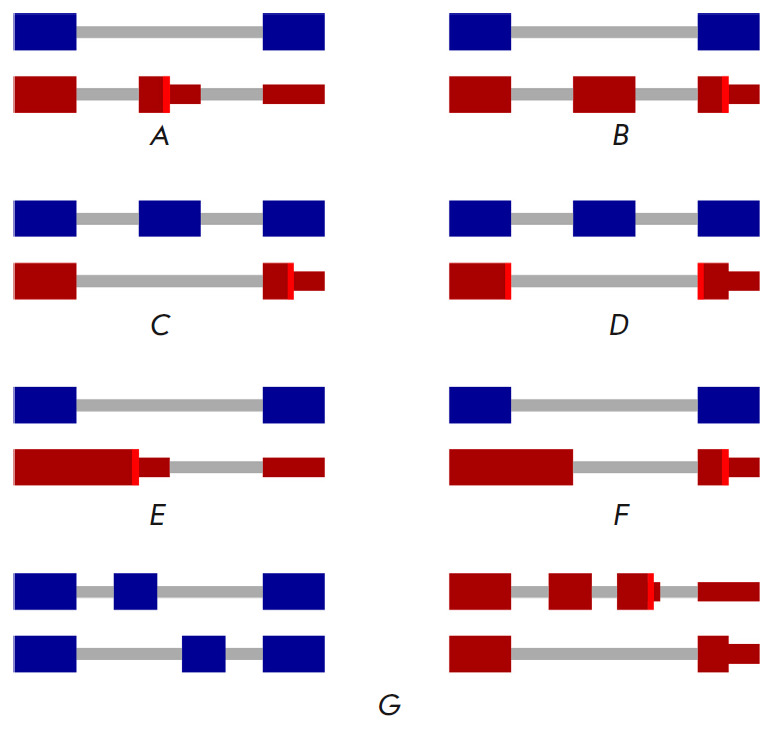
Types of unproductive splicing events. Protein-coding isoforms are shown in blue. Unproductive isoforms are shown in red. PTCs are indicated with bright vertical red lines. (*A*) A poison exon carrying a PTC. (*B*) A poison exon inducing a PTC via frameshift. (*C*) An essential exon inducing a PTC via frameshift. (*D*) An essential exon inducing a PTC on the EEJ. (*E*) An alternative 5’-splice site inducing a PTC via intron retention. (*F*) An alternative 5’-splice site inducing a PTC via frameshift. (*G*) A pair of mutually exclusive exons

AS can generate PTCs in several ways. The best known are the so-called poison exons, which are skipped in the coding isoform but induce a PTC when included in the transcript (*Fig. 2A*) [29, 32, 33]. Poison exons can contain a stop codon within the exon itself or induce a PTC downstream through a frameshift (*Fig. 2B*). The reciprocal case is the so-called essential exon, which is normally included in the coding isoform but induces a PTC when skipped (*Fig. 2C*) [24]. It should be noted that essential exons are usually not a multiple of three in length and cause a frameshift inducing PTCs downstream. However, some essential exons are a multiple of three in length, and the PTC appears at the EEJ formed by their skipping (*Fig. 2D*). Activation of an alternative 5’- or 3’-splice site can also induce a PTC, both due to a frameshift and the formation of a new EEJ (*Fig. 2E,F*). Pairs of mutually exclusive exons can induce a frameshift if both exons are included or both are skipped at the same time (*Fig. 2G*). Thus, PTCs can arise as a result of stop codon insertion at the site of the AS event or somewhere downstream in the transcript.

Of particular interest are splicing events in the 3’-untranslated regions (3’-UTRs). The stop codon preceding the 3’-UTR is not premature; however, splicing of an intron located 50 nts or more downstream creates an NMD target. For example, expression of the AU-rich RNA binding factor AUF1 is regulated by conserved alternatively spliced elements in the 3’-UTR [34]. The 3’-UTRs of transcripts whose expression increases upon inactivation of the NMD system have a larger median length and are enriched in introns [35]. Moreover, most mRNAs encoding NMD factors themselves have long 3’-UTRs and are targets of NMD, which indicates that their expression is autoregulated [35, 36]. Splicing activity in 3’-UTRs significantly increases in tumors, correlates with poor prognosis, and affects many oncogenes [37, 38]. Therefore, unproductive splicing is not limited to premature translation termination within the coding frame and has a remarkable regulatory role in the 3’-UTR.

## ANNOTATION OF UNPRODUCTIVE SPLICING

Current databases contain manually curated and automatically annotated lists of the transcripts that are NMD targets. Tools also exist to systematically classify AS events leading to the generation of NMD isoforms [39].

In databases such as ENSEMBL and GENCODE, NMD targets are annotated using the so-called 50-nt rule. Indeed, the presence of an EEJ 50 nt or more downstream of the stop codon has the greatest predictive power among the features that distinguish NMD transcripts [40, 41]. However, a significant proportion of transcripts that respond to NMD inactivation do not obey this rule [40, 42]. Some genes sensitive to NMD inactivation are annotated as non-coding [40]. According to the data obtained in experiments on NMD inactivation, the presence of upstream open reading frames (uORFs) may be the second most important feature determining the sensitivity of a transcript to NMD [40].

Incompleteness of the existing annotation of NMD transcripts has to do with the fact that their expression levels are normally quite low, hence they fall out of the annotation in databases. Long-read RNA sequencing has shown that many NMD substrates are unstable, and that their expression can be detected at a significant level only when the NMD pathway is inactivated [43]. There is an experimental approach to identifying lowly expressed NMD transcripts, which is based on sequencing of the RNA fraction enriched in EEJ complexes [44]. This fraction contains RNA that is partially spliced but not yet translated. A large number of previously unannotated, conserved EEJs were discovered using this method, with 70% of the exons being not a multiple of three in length and many remaining ones containing stop codons [44].

Unannotated unproductive splicing events can be predicted based on the evolutionary conservation of nucleotide sequences. For example, the *BRD3 *gene contains a conserved intronic region which turns out to be a cryptic poison exon with strong evidence of expression in human tissue transcriptomes [44]. Remarkably, its paralog *BRD2 *also contains a poison exon but in a non-homologous intron, and both these poison exons are surrounded and regulated by conserved RNA structures [44].

## AUTO- AND CROSS-REGULATORY UNPRODUCTIVE SPLICING

Autoregulatory unproductive splicing is often triggered by the accumulation of the gene’s protein product. For example, excess RBM10 protein binds to its own pre-mRNA and induces skipping of two essential exons, which shifts the balance of splice isoforms to NMD targets, and the expression level of RBM10 decreases [45]. This principle governs the expression of many of the genes involved in splicing such as the members of the serine-arginine-rich (SR) gene family [46, 47, 48, 49, 50], *CLK *[51, 52], *TIAL1 *[53],* PTB *[54, 55], *hnRNPD *[56], and some ribosomal proteins [57, 58].

In cross-regulatory unproductive splicing, one protein binds to the pre-mRNA of another and promotes or suppresses NMD isoforms. This type of regulation is also common among RBPs from the SR family [59]. For example, the SRSF3 protein, along with the autoregulatory inclusion of a poison exon in its own pre-mRNA, causes inclusion of poison exons in the transcripts of its paralogs *SRSF2*, *SRSF5*, and *SRSF7* [48]. Besides SR proteins, other pairs of paralogs are regulated in the same way, such as *PTBP1*/*PTBP2* [60], *RBM10*/*RBM5 *[45], *RBFOX2/RBFOX3 *[61],* hnRNPD/hnRNPDL *[56], and *hnRNPL/hnRNPLL *[62]. Generally, cross-regulation among paralogs is a very common phenomenon for RBPs and is characterized by rapid evolutionary dynamics, particularly in regard to acquisition or loss of poison exons [26].

Cross-regulatory unproductive splicing is important not only for RBPs. For example, it causes tissue- specific expression of the *MID1 *gene, which encodes microtubule-associated ubiquitin ligase, whose dysfunction leads to severe embryonic pathologies [27, 63]. Regulated unproductive splicing is important for many physiological processes, such as embryonic development [64], cellular differentiation [65], stress response [66, 67, 68], pathogenesis of neurodegenerative diseases [69, 70], etc.

**Fig. 3:**
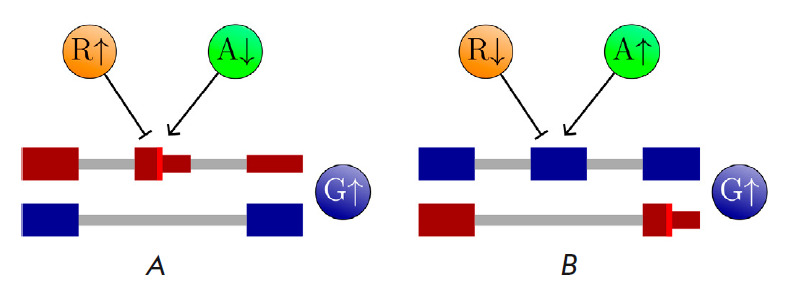
Regulation of unproductive splicing. ‘R’ denotes a splicing repressor. ‘A’ denotes a splicing activator. ‘G’ denotes the target gene. Exon colors are as in *Fig. 2*. (*A*) An increase in R or a decrease in A leads to poison exon skipping, and expression of G increases. (*B*) A decrease in R or an increase in A suppresses essential exon skipping, and expression of G also increases

Both splicing activators and repressors can participate in unproductive splicing regulation. An increase in the concentration of the repressor or a decrease in the concentration of the activator of poison exon inclusion leads to its skipping, thus raising the expression level of the target gene (*Fig. 3A*). Similarly, reduced concentration of the repressor or increased concentration of the activator of an essential exon suppresses its skipping, which also leads to upregulation of the target gene (*Fig. 3B*). It should be noted that some RBPs can serve as both activators and repressors, where the choice between activation and repression depends on the position of their binding site on the mRNA [71]. For example, PTBP1 stimulates

**Fig. 4:**
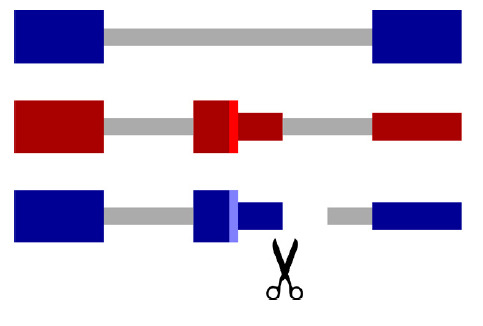
Alternative polyadenylation promotes NMD escape by cutting off a part of the UTR that contains EEJ. This converts a PTC into a normal stop codon (bright vertical blue line)

Many unproductive splicing targets are RBPs, which regulate splicing in other RBPs, creating multiple regulatory loops with positive and negative feedbacks. Negative feedbacks provide autoregulatory mechanisms to maintain homeostasis, while positive feedbacks can create bistable systems to turn on expression [73]. For example, the Drosophila *Sxl *gene employs both these mechanisms for autoinduction at low concentrations and, at the same time, to prevent harmful overproduction of the protein [74]. To achieve such regulation, RBPs can act simultaneously as splicing activators and splicing repressors by binding to multiple sites on the same pre-mRNA. This may explain the high level of evolutionary conservation of nucleotide sequences around unproductive splicing events [75].

## NMD ESCAPE

It was discovered that not all PTCs necessarily cause NMD. A process called NMD escape plays an important role in the pathogenesis of many diseases [76, 77, 78]. NMD escape can be caused by PTC readthrough during translation. The frequency of PTC readthrough depends on the type of the stop codon (UAA, UAG or UGA), and in some NMD-escaping cell populations it can be as high as 20% [79, 80]. NMD escape can also be caused by translation reinitiation [81]. The difference is that translational readthrough produces a full-length protein, while translation reinitiation produces an N-terminal truncated protein and a short C-terminal peptide.

An interesting feature of NMD escape in the human homeostatic iron regulator (HFE) gene is the coordination between NMD and intronic polyadenylation [82]. HFE mRNA contains four alternative polyadenylation sites, one of which mediates NMD escape by pruning the EEJ-containing fragment. Thus, alternative polyadenylation may contribute to NMD escape if premature transcription termination cuts off a part of the untranslated region that contains EEJs, which converts the PTC into a normal stop codon (*Fig. 4*). Transcriptomic studies confirmed that transcripts escaping NMD by alternative polyadenylation are indeed expressed in human tissues [83]. The presence of an intronic polyadenylation site in the human *TAU* gene, which is associated with Alzheimer’s disease, promotes NMD escape [84]. It should be noted that cotranscriptional splicing can prevent premature transcription termination at intronic polyadenylation sites, its functional outcome also being the N-terminal truncated protein [85].

The efficacy of NMD depends on the PTC position in the transcript and other properties. Studies of a large panel of tumor transcriptomes confirmed that the canonical EEJ model is the most important determinant of NMD efficacy [41]. However, the length of the 3’-UTR, proximity to the start codon, the distance between the PTC and the normal stop codon, the length of the exon in which the PTC is located, and other factors have a significant impact. One of them is the RNA structure, which can change the effective distances between cis-elements in the transcript and the binding sites of protein factors such as PABPC1, which apparently has an evolutionarily conserved function in maintaining correct translation termination and counteracting NMD activation [86]. The presence of *cis*-regulatory motifs of splicing factors, such as SRSF1, PABPN1, SNRPB2 and ACO1, also influences the efficacy of NMD [41].

NMD depends on the displacement of EEJ complexes by the ribosome, hence translation control mechanisms may influence its activity. Because miRNAs inhibit translation, they also may affect NMD targets [87], but specific examples of miRNAs that stabilize NMD substrates through this mechanism are currently unknown. In naturally occurring nonsense mutants, microRNAs can, on the contrary, suppress transcripts that escape NMD by binding to the extended 3’-UTR formed after PTC [88]. Interestingly, microRNAs can suppress the activity of the NMD cascade as a whole. For example, the mir-128 microRNA, whose expression level increases in differentiating neuronal cells, suppresses the expression of* UPF1 *and the main component of the EEJ complex* MLN51*, thereby attenuating the response of the NMD system and increasing the expression of the proteins that control neuronal development [89].

## DISEASES ASSOCIATED WITH UNPRODUCTIVE SPLICING AND NMD

Many diseases are associated with malfunctioning of the NMD system and unproductive splicing (*Table 1*). For example, nonsense mutations in the *CFTR *and* hERG *genes cause cystic fibrosis and long QT syndrome, respectively, as a result of the degradation of their transcripts by NMD [90, 91]. Deletions that cause frameshifts also lead to deficiency in important proteins. A well-known example is Duchenne muscular dystrophy, which is often caused by out-of-frame deletions in the *DMD *gene [92, 93, 94].

**Table 1:** Disorders associated with unproductive splicing and NMD

Gene	Disease	Cause, regulators, and therapy	Refs
SCN1A	Dravet syndrome and other epilepsies	SCN1A haploinsufficiency due to mutations, including the ones in intron 20, that promote poison exon inclusion. SSOs switch to the productive isoform (in mouse models)	[95, 96]
SYNGAP1	Autism and mental retardation	Haploinsufficiency of SYNGAP1 due to mutations, including the ones in the splice site. PTBP1 and PTBP2 promote the NMD isoform. SSOs switch to the productive isoform (in mouse models and organoids)	[96, 97]
HTT	Huntington’s disease	Expansion of CAG repeats. Reducing HTT expression by promoting poison exon inclusion using SSOs or small molecules branaplam (NCT05111249) and PTC518 (NCT05358717)	[98]
DMD	Duchenne muscular dystrophy	Frameshift due to a deletion. Eteplirsen (SSO) induces exon 51 skipping to restore the reading frame and to express a truncated but functional dystrophin (FDA approved)	[92, 93]
CFTR	Cystic fibrosis	Nonsense mutation in exon 23. CFTR expression restored by suppressing the inclusion of the mutated coding exon using SSO	[90]
hERG	Long QT syndrome	Nonsense mutation in the penultimate exon. hERG expression restored by retaining the last intron with SSO	[91]
PCCA	Propionic acidemia	Mutation in a cryptic poison exon. HNRNPA1 normally suppresses its inclusion, but the mutation disrupts the HNRNPA1 site and creates a splicing enhancer. PCCA expression restored by switching to a productive isoform using SSO	[99]
FUS	Amyotrophic lateral sclerosis (ALS)	Mutations in the localization signal cause accumulation of FUS in the cytoplasm. FUS suppresses the inclusion of an essential exon in its mRNA, but the autoregulatory loop is disrupted when mutant FUS is localized in the cytoplasm. SSO at the 5’-end of the essential exon switches splicing to unproductive isoform in cell lines	[100]
SNRPB	Cerebro-costomandibular syndrome	Mutations in the poison exon increase its inclusion level and reduce SNRPB expression	[101]
BRD9	Melanoma and other tumors	Mutant SF3B1 promotes poison exon inclusion in BRD9	[102]
EZH2	Myeloid leukemia	Mutant SRSF2 promotes poison exon inclusion in EZH2	[103]
SRSF1	Various tumors	KHDRBS1 switches SRSF1 splicing to the productive isoform	[104]
SRSF3,6,11	Glioblastoma	Increased level of METTL3 leads to the inclusion of the m6A tag in SRSF3,6,11 mRNA and switches their splicing to the productive isoform	[105]
CYR61	Breast cancer	Deregulation of NMD due to hypoxia	[106]
LDHA	Breast cancer	Deregulation of NMD due to hypoxia	[107]

Mutations in splice sites can cause alternative splicing to switch to the NMD isoform. This happens in the *SYNGAP1 *gene whose unproductive splicing is regulated by PTBP1/2 in a tissue-specific manner. Activation of an alternative 3’-splice site generates a NMD target causing the expression level to decrease, thus leading to the development of autism and mental retardation [96, 97].

However, not only mutations in the coding region and splice sites can generate NMD targets. Pathological states can arise due to mutations in introns and non-coding exons, while the mechanism of these pathologies is not always clear. For example, mutations in intron 20 of the *SCN1A *gene promote poison exon inclusion, leading to Dravet syndrome [95, 96]. Mutations in the poison exon of the *SNRPB *gene cause cerebro-costo-mandibular syndrome [101]. It is believed that they create or destroy a binding site of an RBP that activates or suppresses the inclusion of a poison exon, but it currently remains unknown which specific factors regulate these processes. A mutation in the cryptic poison exon of the *PCCA *gene, causing propionic acidemia, is a rare case when the mechanism of unproductive splicing deregulation is known [99]. This mutation is located in the binding site of the HNRNPA factor, which normally suppresses poison exon inclusion, but the mutation destroys this site and simultaneously creates a splicing enhancer, resulting in a decreased *PCCA *expression [99].

Not only mutations splicing cis-elements, but also improper functioning of the regulatory proteins can lead to a disease. A point mutation in the splicing factor SRSF2, which is observed at high frequency in patients with acute myeloid leukemia [103, 108], causes inclusion of a poison exon into histone methylase EZH2 transcripts, leading to its downregulation and, consequently, to the development of myeloid neoplasms, which are normally suppressed by EZH2 [103]. Mutations in the splicing factor SF3B1, which are often observed in myelodysplastic syndromes [109], promote inclusion of a poison exon in the *BRD9* gene, causing a decline in its expression, which results in accelerated growth and metastasis of melanomas [102]. Methylation of the SRSF3, SRSF6, and SRSF11 transcripts due to increased expression of methyltransferase METTL3, which is often observed in glioblastomas, promotes poison exon skipping that causes upregulation of these genes [105]. Remarkably, suppression of METTL3 expression in glioblastoma cell lines reduces cell proliferation and migration by altering splicing of SR protein targets such as BCL-X and NCOR2 [105].

In some cases, pathological changes in unproductive splicing are induced by the tissue condition, while a specific splicing regulator is unknown. For example, hypoxia, which is quite characteristic of many solid tumors, leads to excision of intron 3 from premRNA of the angiogenesis inducer CYR61, a protein promoting cell proliferation and migration in tumors [110, 111, 112]. Under physiological conditions, intron 3 is retained, resulting in expression of the NMD target [106]. Under hypoxia, the activity of the NMD system and regulation through unproductive splicing are disrupted, and CYR61 expression increases, promoting tumor vascularization. Hypoxia also reduces the expression of the alternative isoform of the *LDHA *gene due to unproductive splicing, but the physiological consequence of this decrease is not clear [107].

## MODULATION OF UNPRODUCTIVE SPLICING

Modulation of unproductive splicing is a promising therapeutic strategy for the treatment of many diseases. Splicing can be altered by the so-called splice-switching antisense oligonucleotides (SSO) [113]. The SSOs block the splice sites and/or binding sites of RBPs by complementarily binding to the pre-mRNA sequence and promoting the desired splicing outcome [113].

The SSOs for unproductive splicing modulation can be divided into three groups: SSOs that increase expression of the full-length protein (e.g. by promoting poison exon skipping), SSOs that maintain the expression of a truncated protein when the expression of the full-length protein is impossible (e.g. by promoting exon skipping or intron retention), and SSOs that reduce the expression level (e.g. by promoting poison exon inclusion).

The SSOs belonging to the first group can be used to treat diseases caused by deficiency in a functional protein; for example, due to mutations in genes such as *SYNGAP1*, *SCN1A*, *PCCA*, and *SNRNPB *[95, 96, 97, 99, 101]. SSOs of the second group can be used when a nonsense mutation or a frameshifting deletion renders a PTC, namely to avoid transcript degradation and maintain the expression of the truncated protein. Technically, SSOs of the second group can promote skipping of an exon carrying a nonsense mutation (as in the *PCCA *gene) or retention of an intron downstream of a PTC (as in the *hERG *gene). Coding exon skipping can be useful in the case of a frameshifting deletion to restore the frame (as in the *DMD* gene). A number of SSO drugs for the treatment of Duchenne muscular dystrophy have already been approved [94]. Third-group SSOs can be employed when protein accumulation needs to be suppressed. For example, mutations in the *FUS *gene, which destroy its nuclear localization signal and cause export into the cytoplasm, are associated with amyotrophic lateral sclerosis [114, 115]. To suppress the expression of *FUS *via unproductive splicing, its protein product must be located in the nucleus; however, the export of the mutant protein from the nucleus destroys the autoregulation loop, which aggravates its accumulation in the cytoplasm and promotes the formation of aggregates exhibiting cytotoxic effects [100, 116, 117, 118].

Despite all the positive aspects of SSO, there are many difficulties related to their delivery to target organs and tissues. The need to develop a delivery system and high drug doses in order to achieve the required concentration lead to higher prices and an increased risk of side effects [119]. Small molecule splicing modulators, which are more bioavailable, offer a powerful alternative to SSO.

A number of low-molecular-weight compounds that bind to splicing factors are currently known; however, they simultaneously modulate splicing of many genes [119]. Several small molecules have been found that specifically bind to target RNAs [98, 119, 120, 121, 122]. The best studied of these, risdiplam, modulates splicing of the *SMN2 *gene and can be used to treat spinal muscular atrophy [121]. Branaplam, similar in structure and mechanism of action to risdiplam, promotes the inclusion of the cryptic poison exon in the *HTT *gene, which reduces its expression and slows down the progression of Huntington’s disease [122]. The small molecule PTC518, which is currently in phase 2 clinical trials, has a similar effect [120].

## CONCLUSION

Unproductive splicing, an evolutionarily conserved mechanism of post-transcriptional regulation of gene expression, arose as a result of interaction between alternative splicing and nonsense-mediated decay. Unproductive splicing intricately maintains the balance of gene expression levels through the auto- and cross-regulatory cascades containing both positive and negative feedback loops. It is closely related to many other cellular processes, such as intronic polyadenylation, regulation of translation, and interactions with microRNAs. Deregulation of unproductive splicing is the cause of many human diseases, for which splice-switching antisense oligonucleotides offer a promising therapeutic strategy.

## Abbreviations

NMD: Nonsense-mediated decay
PTC: premature termination codon
EEJ: exon- exon junction
AS: alternative splicing
RBP: RNA-binding protein
UTR: untranslated region
SSO: splice-switching antisense oligonucleotides
nt: nucleotide
